# 480. Duration of Infectious Virus Shedding by SARS-CoV-2 Omicron Variant among Immunocompromised Patients

**DOI:** 10.1093/ofid/ofad500.550

**Published:** 2023-11-27

**Authors:** K O H E I KAMEGAI, Noriko Iwamoto, Naoya Itoh, Masahiro Ishikane, Yusuke Asai, Nana Akazawa, Noriko Fuwa, Jin Takasaki, Masayuki Hojo, Akira Hangaishi, Tomiteru Togano, Katsuji Teruya, Takayuki Kanno, Kenichiro Takahashi, Sho Miyamoto, Yuichiro Hirata, Tomoya Saito, Harutaka Katano, Tadaki Suzuki, Norio Ohmagari

**Affiliations:** National Center for Global Health and Medicine, Shinjuku, Tokyo, Japan; Disease Control and Prevention Center, Tokyo, Tokyo, Japan; Aichi Cancer Center, Nagoya, Aichi, Japan; Center Hospital of the National Center for Global Health and Medicine, Tokyo, Tokyo, Japan; National Center for Global Health and Medicine, Shinjuku, Tokyo, Japan; Aichi Cancer Center, Nagoya, Aichi, Japan; National Center for Global Health and Medicine, Shinjuku, Tokyo, Japan; National Center for Global Health and Medicine, Shinjuku, Tokyo, Japan; Center Hospital of the National Center for Global Health and Medicine, Tokyo, Tokyo, Japan; Department of Hematology, Shinjuku, Tokyo, Japan; National Center for Global Health and Medicine, Shinjuku, Tokyo, Japan; Center Hospital of the National Center for Global Health and Medicine, Tokyo, Tokyo, Japan; National Institute of Infectious Diseases, Shinjuku, Tokyo, Japan; National Institute of Infectious Diseases, Shinjuku, Tokyo, Japan; National Institute of Infectious Diseases, Shinjuku, Tokyo, Japan; National Institute of Infectious Diseases, Shinjuku, Tokyo, Japan; National Institute of Infectious Diseases, Shinjuku, Tokyo, Japan; National Institute of Infectious Diseases, Shinjuku, Tokyo, Japan; National Institute of Infectious Diseases, Shinjuku, Tokyo, Japan; National Centre for Global Health and Medicine, Shinjuku, Tokyo, Japan

## Abstract

**Background:**

Immunocompromised patients with coronavirus disease (COVID-19) shed SARS-CoV-2 RNA longer than usual, but the duration of viral shedding and criteria for nosocomial de-isolation in these patients remains unclear.

**Methods:**

A prospective cohort study was performed at two tertiary medical centers in Japan during the Omicron epidemic waves from July 8, 2022, to January 30, 2023. Nasopharyngeal swab samples were serially collected from immunocompromised COVID-19 patients. We included populations with either hematological malignancies, solid tumors, autoimmune diseases, or human immunodeficiency virus infection. Patients were classified as severely or moderately immunocompromised based on the national COVID-19 guidelines in Japan. The relationship among patients' characteristics, immune status, quantitative reverse transcription PCR (qRT-PCR), and duration of infectious virus shedding between the two groups was assessed using Mann-Whitney U and Fisher's exact tests.

**Results:**

Among 41 patients with 163 samples, nine patients and 47 samples were severely immunocompromised, and 32 patients and 116 samples were moderately immunocompromised. The median ages of the two groups were 70 (IQR: 56-71) and 70.5 (IQR: 60.75-75) years, respectively.

In the severely immunocompromised group, 87.2% of the samples (41/47) were PCR-positive, with a median Cq value of 26.1 (IQR: 20.5-30.2), and 36.2% (17/47) were culture-positive. In the moderately immunocompromised group, 75.0% of the samples (87/116) were PCR-positive, with a median Cq value of 21.9 (IQR: 18.4-28.6), and 38.8% (45/116) were culture-positive (Table 1).

Of the 62 culture-positive samples, five (8.1%) were culture-positive from day 20 onwards, all from severely immunocompromised patients (P = 0.001). In moderately immunocompromised patients, none were culture-positive on day 12 or later (Figure 1).

**Figure:**
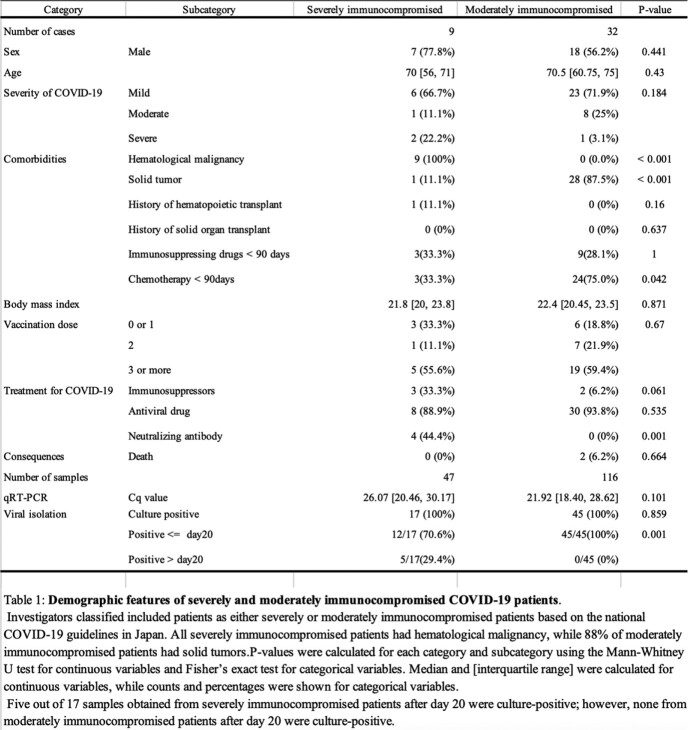

**Figure:**
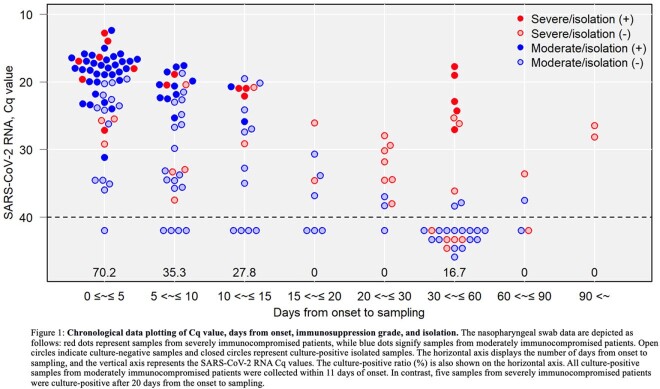

**Conclusion:**

In COVID-19 patients, particularly those severely immunocompromised, the duration of viral shedding should be closely monitored. Further research is needed to establish a safe period for in-hospital de-isolation of immunocompromised patients.

**Disclosures:**

**All Authors**: No reported disclosures

